# Summer Diatom Blooms in the North Pacific Subtropical Gyre: 2008–2009

**DOI:** 10.1371/journal.pone.0033109

**Published:** 2012-04-06

**Authors:** Tracy A. Villareal, Colbi G. Brown, Mark A. Brzezinski, Jeffrey W. Krause, Cara Wilson

**Affiliations:** 1 Marine Science Institute and the Department of Marine Science, The University of Texas at Austin, Port Aransas, Texas, United States of America; 2 Marine Science Institute, University of California Santa Barbara, Santa Barbara, California, United States of America; 3 Department of Ecology, Evolution, and Marine Biology, University of California Santa Barbara, Santa Barbara, California, United States of America; 4 Environmental Research Division, NOAA/NMFS Southwest Fisheries Science Center, Pacific Grove, California, United States of America; The University of Texas at Austin, United States

## Abstract

The summertime North Pacific subtropical gyre has widespread phytoplankton blooms between Hawaii and the subtropical front (∼30°N) that appear as chlorophyll (chl) increases in satellite ocean color data. Nitrogen-fixing diatom symbioses (diatom-diazotroph associations: DDAs) often increase 10^2^–10^3^ fold in these blooms and contribute to elevated export flux. In 2008 and 2009, two cruises targeted satellite chlorophyll blooms to examine DDA species abundance, chlorophyll concentration, biogenic silica concentration, and hydrography. Generalized observations that DDA blooms occur when the mixed layer depth is < 70 m are supported, but there is no consistent relationship between mixed layer depth, bloom intensity, or composition; regional blooms between 22–34°N occur within a broader temperature range (21–26°C) than previously reported. In both years, the *Hemiaulus-Richelia* and *Rhizosolenia-Richelia* DDAs increased 10^2^–10^3^ over background concentrations within satellite-defined bloom features. The two years share a common trend of *Hemiaulus* dominance of the DDAs and substantial increases in the >10 µm chl *a* fraction (∼40–90+% of total chl *a*). Integrated diatom abundance varied 10-fold over <10 km. Biogenic silica concentration tracked diatom abundance, was dominated by the >10 µm size fraction, and increased up to 5-fold in the blooms. The two years differed in the magnitude of the surface chl a increase (2009>2008), the abundance of pennate diatoms within the bloom (2009>2008), and the substantially greater mixed layer depth in 2009. Only the 2009 bloom had sufficient chl a in the >10 µm fraction to produce the observed ocean color chl increase. Blooms had high spatial variability; ocean color images likely average over numerous small events over time and space scales that exceed the individual event scale. Summertime DDA export flux noted at the Hawaii time-series Sta. ALOHA is probably a generalized feature of the eastern N. Pacific north to the subtropical front.

## Introduction

The euphotic zone of the North Pacific subtropical gyre (NPSG) is a low nutrient, low autotrophic biomass environment long thought to be monotonic and dominated by small (<5 µm diameter) prokaryotes. However, the advent of satellite-borne sensors for remote measurement of ocean color has revealed extensive chlorophyll (chl) blooms in the summertime NPSG [1] that appear to be concurrent with blooms of nitrogen-fixing diatom symbioses at both the Hawaii Ocean Time-Series Station ALOHA (22.75°N, 158°W), and at the subtropical front (∼30°N) north and east of Hawaii [2], [3], [4], [5], [6]. These diatom symbioses (DDAs, diatom-diazotrophic associations) are one component of the diverse oceanic diazotroph community [7], [8] and are comprised of a diatom host and either an endosymbiotic or exosymbiotic heterocystous cyanobacteria. The most commonly reported DDAs consist of the endosymbiont *Richelia intracellularis* Schmidt and the hosts *Rhizosolenia* and *Hemiaulus* or the exosymbiont *Calothrix rhizosoleniae* Karsten with *Chaetoceros* or *Bacteriastrum* spp. [8]. The symbioses are distributed tropically and sub-tropically in all oceans at varying abundance [9], [10] and often require epifluorescent microscopy to visualize [11], [12].

Diazotrophy is an important nitrogen source in the nutrient-limited NPSG ecosystem and is considered to be a likely source supporting the summer chl blooms. N_2_-fixation rates can at times equal or exceed nitrate flux across the nutricline [13], [14] and, unlike nitrate, supports net drawdown of atmospheric CO_2_ [15]. Thus, the biogeochemical role of diazotrophs in the system varies with species-specific properties such as aggregation, sinking rate and susceptibility to grazing. The *Hemiaulus* DDA forms large aggregates in blooms [2] and is an important source for the annual summer carbon export at Sta. ALOHA [16], [17].

The unpredictability of the DDA blooms, their distance from shore, and the logistic lead-time required to sample them, particularly when they occur at the subtropical front (STF), has resulted in significant under-sampling and the frequent use of proxies to understand their dynamics. The source of nutrients to sustain observed chl blooms has been unclear, and it has been assumed that increases in diazotrophs are important [1], [4], [5]. In general, NPSG observations at Sta. ALOHA suggest blooms of large diazotrophs (*Trichodesmium* and DDAs) are restricted to the June-October period, surface water temperatures from 25–27°C, and mixed layer depths (MLD) <70 m [5], [6]. High abundance of the diazotrophic symbiont *R. intracellularis* and *Trichodesmium* do not appear to co-occur [6]. Blooms rich in DDAs at the STF also occur during summer months (June-October), when the water column is well stratified, but the relationships to MLD and temperature are not well constrained. The filamentous, free-living cyanobacteria *Trichodesmium* spp. and coccoid diazotrophs are typically the most abundant taxa during blooms of diazotrophs at Station ALOHA [6], [18] but mesoscale features can result in elevated Het-1, 2 or 3 *nifH* copy counts diagnostic of DDA increases [18], [19]. In these cases, DDA blooms are often concurrent with summertime chl blooms visible in satellite ocean and appear wrapped around the periphery of eddies [4], [20], linked with eddy passage at Station ALOHA [5], [6], [19], or associated with the STF [2], [3], [4]. They can also occur southwest of Hawaii in areas subject to frequent eddy development [21].

A linkage between DDA blooms and mesoscale oceanic features is implied, but has been difficult to explain mechanistically since many of the more northerly blooms do not co-occur with sea surface height (SSH) anomalies [1]. A relationship to nutrient injection is implied both by the link of the northerly blooms to critical latitude internal wave propagation [22] and modeling studies that suggest frontogenesis in regions of large horizontal stretching associated with mesoscale features [20], [23]. Fe inputs will likely stimulate blooms as well [23]; a more southerly chl bloom was evident in 2010 ocean color data as far east as 168°E [23] and was linked to dust inputs, seasonal temperature increases and frontal upwelling.

The difficulty in linking satellite and field observations is evident in that in DDA blooms identified by direct cell counts have been observed as far west as 170°W without a distinctive ocean color signature [2]. Both indirect [6] and direct observations [2] have challenged the assumption of a DDA origin of the satellite bloom signature, noting that pigment increases in the DDA size fraction (>5 or >10 µm) could not account for the observed chl signatures nor did high abundance DDA blooms give consistent ocean color signatures. Although abundant evidence exists for periodic *Hemiaulus* blooms in the N. Pacific [5], the role DDAs play in creating the observed satellite signature or biomass increase remains unresolved [2], [6], as does the relationship to other phytoplankton species and the similarity of blooms between regions of the N. Pacific [2]. For example, the non-symbiotic pennate diatoms, *Mastogloia* spp., can co-exist with DDAs and even exceed DDA abundance [2], but direct enumeration of these diatom communities by microscopy is rare.

Here we report the results of focused sampling of summer chlorophyll blooms in the NPSG during 2008 and 2009. Our goal was to quantitatively enumerate the diatom flora in blooms during both years, examine spatial gradients in abundance, examine relationships between the netplankton fraction and ocean color signatures, and document changes in dominant species relative to chl a increases. By combining this data and previously reported diatom counts, we were able to generalize our results across multiple years and expand the known temperature range and environmental conditions associated with the DDA component of the blooms.

## Methods

**Figure 1:**
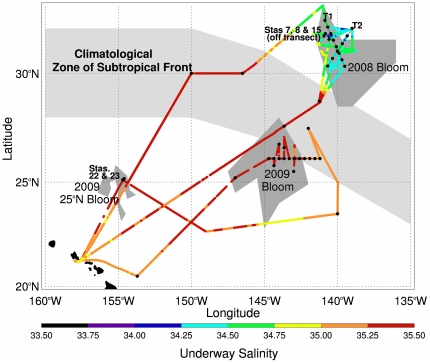
Cruise tracks for 2008 and 2009. Surface salinity data from the ship’s underway sampling system is color coded along the shiptrack. The climatological mean position of the subtropical front [48], [49] is shaded in light grey. Bloom areas were derived from ocean color data (see text for details).

Two cruises were conducted in the NPSG (2008/2009 *R/V Kilo Moana*) to sample satellite ocean color features for phytoplankton biomass and species composition (Fig. 1). In 2008, two transects were sampled across a single event. In 2009, two features were sampled, one at 30°N, 138°W region with a second feature sampled at 25°N, 154°W (Fig. 1). Data from each year are presented separately in the Results.

Ocean color data were provided daily from the Moderate Resolution Imaging Spectroradiometer (MODIS) sensor on NASA's Aqua satellite. The west coast regional node (WCRN) of NOAA’s Coast Watch program processed the NASA data stream and sent it to the ship daily. The data are available at 2.7 km resolution for the Pacific basin, and is mapped to an equal angle grid that is 0.025° latitude by 0.025° longitude using simple arithmetic means to produce composite daily images. Monthly composites of satellite SST and satellite chlorophyll data were used to examine the SST associated with the 30°N chlorophyll blooms. The SST data used was the Pathfinder Version 5.0 Sea Surface Temperature (SST) data set from NOAA's Advanced Very High Resolution Radiometer (AVHRR) [24], [25]. Note that in the text we refer to ocean color-derived chlorophyll as chl. Analytically-determined chlorophyll from fluorometry is termed chl a.

Seawater for nutrient analysis [26], formalin-preserved [27] quantitative phytoplankton counts (transmitted light inverted microscopy, Zeiss ICM-405, 50 ml settled volume) [28], epifluorescent (Olympus BX51, excitation 480 nm/emmission >520 nm) symbiont counts on filters [29], chlorophyll *a* (MeOH extraction, non-acidification method [30], and biogenic silica (bSi) was collected from a Seabird 911 CTD with a 24 Niskin bottle (12-L) rosette. Unfiltered seawater was frozen and analyzed ashore on a LaChat Quikchem 8000. Detection limits were 0.05–0.1 µmol L^−1^ for nitrate+nitrite, silicate and phosphate. 50-mL samples were also analyzed at sea for silicate using a more sensitive colorimetric method [31]. Due to time constraints, not all measurements could be made at each station. Vertical net tows (35 µm mesh) in the upper 50 m were also used to collect phytoplankton for visualization and quantification.

In the settled phytoplankton counts, small pennate diatoms were routinely observed using light microscopy; scanning electron microscopy from 2009 samples showed *Mastogloia woodiana* Taylor dominated the samples. Other pennate diatoms were present at various subdominant abundance; *M. woodiana* will be used to represent this group in general throughout the text. Net tow samples were examined under transmitted and epifluorescence illumination at sea to confirm the presence of symbionts associated with large diatoms [12].

*Hemiaulus hauckii* symbiont enumeration was complicated by the occurrence of two types of symbiont fluorescence. In contrast to the typical orange/yellow fluorescing *Richelia* symbionts seen in *H. membranaceus* Cleve [12] at stations to the south of the STF on these cruises, the *Hemiaulus hauckii* symbiont in the 30°NPSG blooms was only observed as red-fluorescing trichomes in examination of living net-collected material at sea. The red-fluorescing trichomes were not visible at all in diatom cells on the 10 µm pore size filters due to optical interference by the filters giving the appearance of no symbionts (orange fluorescence is a diagnostic marker for the cyanobacterium’s phycobilins); however, they were visible in net-tow collected samples examined on glass slides at sea (∼2 heterocysts cell^-1^). The cells usually had to be slightly crushed on the slide and the contents teased apart to see the symbiont, but they were visible within 99+% of the cells and could be recognized in some cells without manipulation [2]. *Richelia* symbionts in *Rhizosolenia* and *H. membranaceus* fluoresced orange and were readily visible on the filters (termed golden *Richelia* throughout the text to differentiate them from the red-fluorescing *Hemiaulus hauckii* Grunow in Van Heurck symbiont). *H. membranaceus* (<50 cells L^−1^ in 2008) was numerically rare in the samples from the blooms and is not included in the data plots. 

Chlorophyll *a* (chl a ) was size-fractionated by filtering through 47 mm Isopore membrane filters of either 0.4 µm (250 ml) or 10 µm pore size filters (500 ml) in duplicate for each pore size. For the measurement of biogenic silica (bSi), duplicate 2.8 L subsamples of seawater were filtered through either a 47-mm 0.6 µm and 10 µm pore polycarbonate filters, dried, and analyzed in the laboratory using a alkaline digestion method using Teflon tubes that produced lower and more stable blank values [32]. Water-column integrations of particulate concentrations and diatom abundances were derived from trapezoidal integration of the data. Retrospective analysis of cell abundance and chlorophyll data used previously published datasets [2], [33].

Mixed layer depth (MLD) was determined at each station from the CTD data using 1-m binned temperature and salinity data to construct profiles of water density. The depth of the mixed layer was defined as the depth where density increased by 0.125 kg m^−^^3^ relative to surface values [34]. Units follow SI conventions and standardized to m^−3^ except where standard oceanographic usage refers to L^−1^ (e.g. cell counts, nutrients, chl, bSi).

## Results

The sampling captured different temporal components of the DDA events in each year. A declining bloom was sampled in 2008 and two blooms features were sampled in 2009: one that had ended by the time of occupation and one that was sampled shortly after its appearance. Each will be presented separately prior to discussing their similarities and differences. We use an ocean color chl bloom definition of >0.15 µg L^−1^ [1] and a DDA bloom of >1000 cells L^−1^ [2]. The two metrics measure different properties and cannot be used interchangeably. Both are operational definitions. The results from 2008/2009 are generalized by including previously published data sets in the analysis from cruises in 1995, 2002 and 2003 [2], [33]. In the Discussion, a broader perspective on the thermal range for blooms is derived from a multiple year comparisons of satellite chl blooms and temperature along a latitudinal gradient.

### 2008 Bloom

The 2008 chl feature was centered at 140°W and became visible in satellite imagery the day the R/V *Kilo Moana* left port (1 July 2008). It was past its peak by the time the shipboard sampling commenced (7 July 2008, Fig. 2a, b). This feature occurred at and around the STF’s characteristic 34.8 isohaline [35] (Fig. 1), and was superimposed on a complex salinity structure (Fig. S1, S2, S3). Surface salinity values varied from 33.6–34.6 PSU over 1–3 days in the bloom region (Fig. 1) while temperature ranged from 19.7–21.5°C. Surface nitrate+nitrite and phosphate concentrations were below detection limits above the nutricline (Fig. S1, S2, S3).; silicate was at least two orders of magnitude higher than the detection limits for inorganic N and P. The mixed-layer results are presented in a later section when multiple cruise comparisons are made.

**Figure 2:**
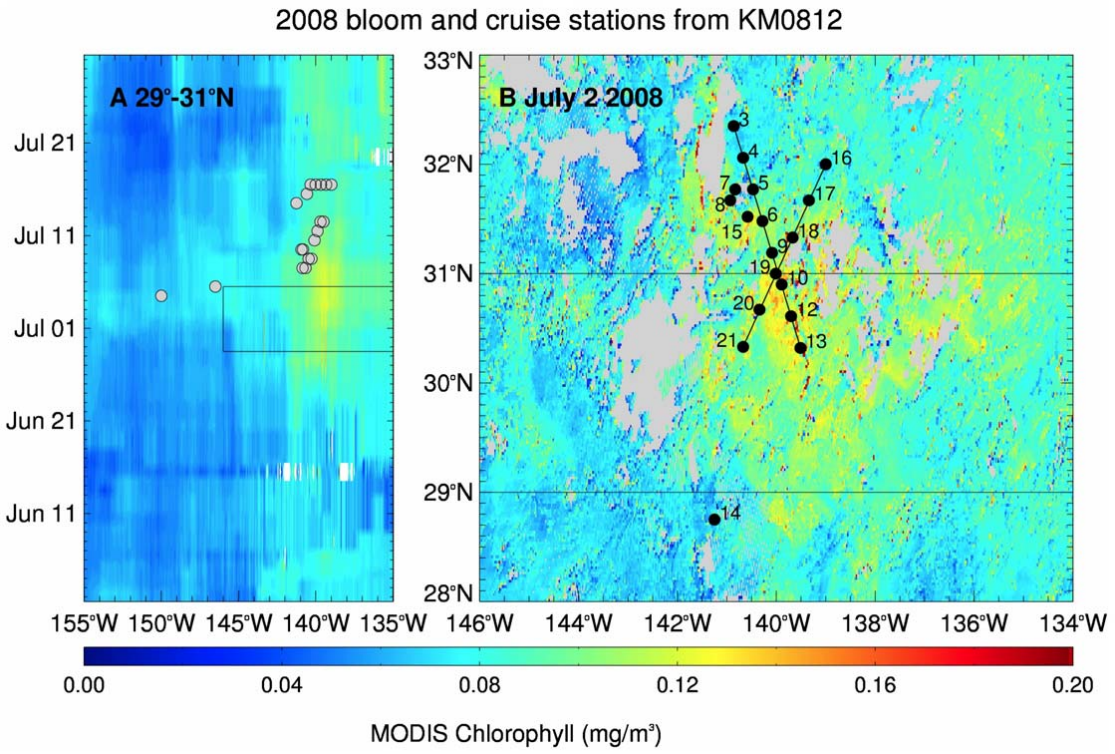
Satellite chlorophyll *a* estimate with overlying stations for 2008. (a) Hovmöller diagram of MODIS chl showing the development of the 2008 bloom and (b) an 8-day composite showing the maximum extent of the chlorophyll bloom, overlaid with station locations. The black lines on (a) depict the temporal and longitudinal extent of the data shown in (b), and the black lines on (b) depict the latitude range of the data shown in (a). The image is an 8 day composite centered on July 3, 2008. Stas. 1 and 2 (see Table 1) are off the map. Transect 1 (Sta 3–15), Transect 2 (Sta. 16–21).

**Table 1:** Station information, 0–60 m integrated data (m^−2^) and surface chlorophyll (µg L^−1^) from 2008.

Sta.	Lat. (°N)	Lon. (°W)	H. hauckii (106 cells m^−2^)	Rhizosolenia (106 cells m^−2^)	M. woodiana (106 cells m^−2^)	Chaetoceros sp. (106 cells m^−2^)	R.intracellularis (Filaments m^−2^)	Calothrix (Filaments m^−2^)	Chl a (mg m^−2^)	>10 µm Chl a (mg m^−2^)	%>10 µm Chl a	bSi (mmol m^−2^)	Surface Chl a (µg L^−1^)
1	30.00	150.00	4.6	0.8	1.5	0	0.2	0	4.59	0.54	11.83	1.31	0.06
2	30.00	146.30	9.7	0.2	3.4	3.4	0.6	0	4.64	0.61	13.04	4.09	0.04
3	32.21	140.51	10	0.2	2.2	0.08	0.2	0	6.53	0.65	9.98	4.79	0.09
4	32.03	140.40	–	–	–	–	–	–	6.33	0.90	14.17	6.36	0.07
5	31.46	140.28	11.5	1.2	0.9	0	0.1	0	5.76	0.62	10.78	4.42	0.08
6	31.28	140.16	–	–	–	–	–	–	5.58	0.78	13.98	2.44	0.09
7	31.46	140.49	117	4.8	10.6	1.0	0.4	0	6.28	1.44	22.95	9.41	0.10
8	31.40	140.55	–	–	–	–	–	–	6.44	1.01	15.71	3.95	0.08
9	31.11	140.04	11.8	0.3	3.0	0	0.09	0	4.72	0.71	15.03	3.78	0.08
10	30.53	139.53	–	–	–	–	–	–	6.28	0.68	10.76	3.09	0.07
12	30.36	139.42	4.9	0	1.0	0	0.05	0	4.41	0.61	13.88	2.91	0.06
13	30.19	139.30	–	–	–	–	–	–	5.17	0.67	12.98	2.57	0.06
14	28.45	141.15	6.6	0.2	2.6	0	0.5	0	4.74	0.78	16.47	2.21	0.07
15	31.31	140.34	–	–	–	–	0.1	0	5.49	0.73	13.20	3.78	0.08
16	32.00	138.59	2.0	0	0.9	0	0.2	0	4.87	0.66	13.47	1.85	0.07
17	31.40	139.19	0.3	0	0.4	0	–	–	5.88	0.69	11.71	1.41	0.09
18	31.19	139.40	13.1	0	0.4	0	–	–	6.14	0.82	13.43	2.07	0.09
19	30.59	140.00	28.6	0	1.3	0	–	–	6.14	0.87	14.13	3.21	0.08
20	30.40	140.20	13.1	0	0.5	0	–	–	5.85	0.77	13.08	1.10	0.08
21	30.19	140.40	5.4	0	0	0.4	–	–	5.49	0.62	11.36	N/A	0.04

**Figure 3:**
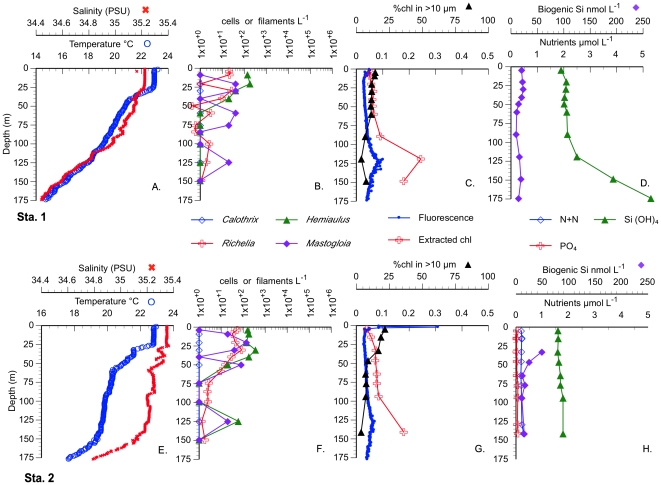
Vertical profiles of Sta. 1 and 2 (outside the bloom area) in 2008. A-D Station 1, E-H Sta. 2. A, E. Temperature and Salinity CTD profiles. B, F. Cell and symbiont abundance. C., G. Chl *a*. (CTD fluorescence, extracted and %>10 µm). D., H. Silicate and biogenic silica concentrations.

**Figure 4:**
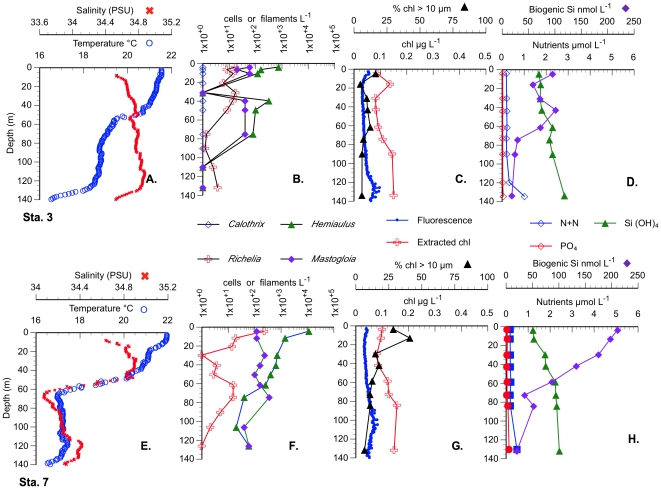
Comparison of two stations within the 2008 chl bloom. Vertical profiles of Sta. 3 &7 in 2008. A-D Station 3 (representative station), E-H Sta. 7 (highest abundance station). A, E. Temperature and Salinity CTD profiles. B, F. Cell and symbiont counts. C., G. Chl *a*. (CTD fluorescence, extracted and %>10 µm). D., H. Nutrient and biogenic silica concentrations.

Non-bloom stations (Stas. 1 and 2), sampled en route and located south and west of the bloom (Table 1 for location), were typical gyre stations with shallow mixed layers (∼25 m) and low nutrient concentrations (Fig. 3). Individual diatom spp. abundance at these stations was ≤2×10^2^ cells L^−1^, golden *Richelia* abundance was <100 trichomes L^−1^, bSi was low (10–50 nmol L^−1^), and chl a in the >10 µm fraction was ≤10% (Fig. 3). The targeted chl feature (Fig. 2b, Sta. 3–21) did not reach the >0.15 mg m^−3^ bloom threshold; nevertheless, the chlorophyll values are clearly elevated relative to the surrounding ocean (Fig. 2). DDA abundance was <∼2.0–5.0×10^2^ cells L^−1^ in all but 3 of the stations ((Fig. S1, S2, S3).). The highest DDA abundance was noted at Station 7 where *Hemiaulus* reached >11,000 cells L^−1^ at the surface; Fig. 4). This station also had the highest bSi concurrent with dissolved silicate drawdown (relative to other stations) and the greatest proportion of chl a in the >10 µm size fraction (40%). At all stations, DDA host taxa increases were maximal in the upper 25–30 m with abundance decreasing rapidly with depth (Fig. 4, (Fig. S1, S2, S3). Integrated cell abundance (0–60 m; Table 1) varied an order of magnitude over 10s km as did chl a and bSi; red-fluorescing symbionts in *Hemiaulus* dominated the total DDAs. Orange-fluorescing *Richelia* also increased at Station 7 (Fig. 4), but integrated abundance was nearly 50% higher at Sta. 2 580 km southwest of Sta. 7 (*see* Figs. 3, 4; Table 1). Non-symbiotic diatom species co-occurred with the DDA species at all stations and also had elevated cell abundance at bloom Station 7, although they did not demonstrate the multiple order of magnitude increase noted for *Hemiaulus* (Figs. 3, 4; Table 1; (Fig. S1, S2, S3). The *Mastogloia woodiana* abundance maximum at Sta. 7 was subsurface and was distinct from the *H. hauckii* primary maxima at the surface (Fig. S1, S2, S3).

Maximum surface chl a concentration was at Station 7 (0.10 µg L^−1^), but was only slightly elevated over the surface concentrations (0.07–0.10 µg L^−1^) at other stations (Table 1)**.** The deep chlorophyll maximum (DCM) depth was uniform across the two transects (Fig. S1, S2, S3). The >10 µm chl a surface values were low in the two transects (0.01 µg L^−1^) with 15–20% of total chl a in the %> 10 µm fraction (Fig. S1, S2, S3). and increasing at Station 7 to ∼40% of the total extracted chl a at the surface concurrent with the DDA cell maximum (Fig. 4; Fig. S1, S2, S3). While Sta. 7 had the highest surface diatom abundance, depth-integrated chl a (both size fractions) and % chlorophyll in the >10 µm size fraction were greater at other stations (Table 1).

The >10.0 µm size fraction of [bSi] represented >80%, on average, of the total (0.6 µm) size fraction (r^2^ = 0.79); therefore, only the 0.6-µm-size fraction data are presented. [bSi] maxima were associated with the local diatom cell maxima on each transect, with the maximum value (211 nmol L^−1^) found in the Sta. 7 surface sample (Fig. 4; Fig. S1, S2, S3). Outside of Sta. 7 but within the bloom region, [bSi] varied around a mean of ∼50 nmol L^−1^, two-fold higher than [bSi] observed in other parts of the gyre [33], [36] and at Sta. 1 ∼900 km to the west-southwest (<25 nmol L^−1^; 30.00 N 150.00 W). Integrated [bSi] for the bloom area (Table 1) was also generally higher than observed in more southerly regions of the gyre (e.g. [33], [36]).

### 2009 Post-Bloom

**Figure 5:**
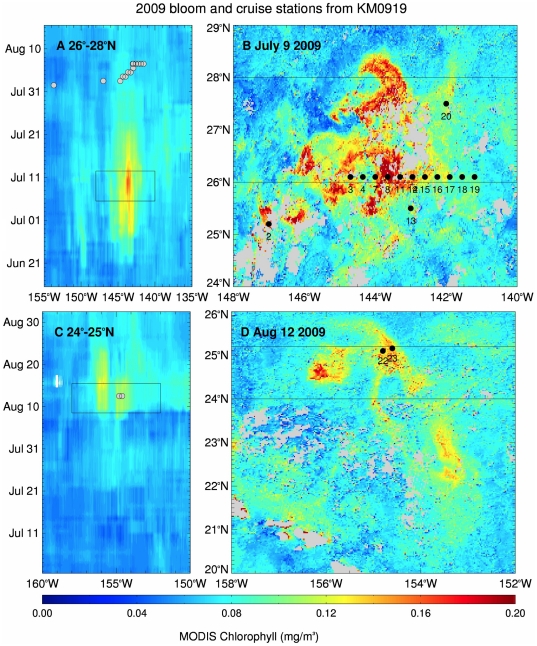
Satellite chlorophyll *a* estimate with overlying stations for 2009. (a) Hovmöller diagram of MODIS chl showing the development of the 2009 bloom at 140°W and (b) an 8-day composite showing the maximum extent of the chlorophyll bloom at 140°W, overlaid with station locations. (c) Hovmöller diagram of MODIS chl showing the development of the 2009 bloom at 155°W and (d) an 8-day composite showing the maximum extent of the chlorophyll bloom at 155°W, overlaid with station locations. The black lines on (a) and (c) depict the temporal and longitudinal extent of the data shown in (b) and (d), and the black lines on (b) and (d) depict the latitude range of the data shown in (a) and (b).

An ocean color chlorophyll bloom developed unusually early [28], [31] at 144°W in late June 2009. By the time the ship arrived in mid-July, the bloom had largely disappeared (Fig. 1, 5); we refer to this as the post-bloom or the 144°W bloom. During the cruise, another ocean color chlorophyll feature developed at 25°N 155°W (Fig. 2b, c) and was sampled near the end of the cruise (Sta. 22, 23); this feature will be referred to as the 2009 25°N bloom and is discussed in the next section.

The post-bloom region was located south of the climatological mean STF position (Fig. 1). While there was no distinctive surface salinity signature of the subtropical front, the subsurface salinity structure was highly variable and consistent with front characteristics (Wilson et al., unpublished). Surface water temperature was 25.6–25.9°C in the post-bloom area. Nitrate+nitrite and phosphate were below detection limits in the upper 150 m; silicate was depleted relative to the 2008 bloom area, with surface values of 1.00–1.75 µM increasing to 2.25 at >120 m (Figure S4).

A transect was conducted along 26°N, between 141–145°W, where the bloom had been observed with satellite data several weeks previously (Fig. 5a). Diatom abundance was low (Fig. S4) with ∼10^1^ cells L^−1^ for *H. hauckii,* golden *Richelia,* and *M. woodiana.* The *Chaetoceros*/*Calothrix* DDA was not observed in the 2009 post-bloom. Surface chl a concentrations were very low (∼0.05 µg chl a L^−1^ total chl) and with ≤10% of the total chl a in the >10-µm fraction (Fig. S4). [bSi] was the lowest seen in the two cruises at ≤20 nmol L^−1^. Integrated values abundance, size-fractionated chl a and bSi all indicate a biologically impoverished water column in this region.

### 2009 25°N bloom

**Figure 6:**
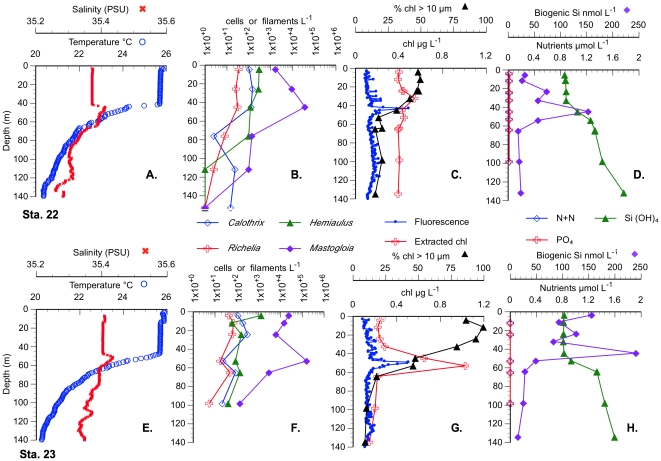
Station 22 (A-D) and 23 (E-H) on 12 Aug. 2009. A, E. Temperature and Salinity CTD profiles. B, F. Cell and symbiont abundance. C., G. Chl a values. D., H. Nutrient and biogenic silica concentrations.

The passage of hurricane Felicia to the south delayed the departure from the post bloom area resulting in only two stations being occupied in an intensifying feature at 25°N, 155°W (Fig. 5). This bloom feature was located well to the north of the hurricane’s path and was unaffected by its passage. The 50 m mixed layer was typical of gyre interior conditions at this time of the year (T = 25.1°C, S = 35.3–35.4). Nutrient concentrations above 140 m were below detection limits with the exception of silicate (Fig. 6), which was the lowest sampled during the 2009 cruise; the nutricline depth was >100 m.

Surface chl a concentration was elevated at both of the 25° bloom stations (Sta 22, 0.40 µg L^−1^; Sta. 23, 0.25 µg L^−1^; Fig. 6; Table 2). Diatom abundance (*H. hauckii, Rhizosolenia* spp., *M. woodiana,* and *Chaetoceros* spp) was highest at Sta. 23 and exceeded 10^5^ cells L^−1^ (Fig. 6); DDAs had a near-surface maximum. The pennate diatom complex *M. woodiana* was numerically dominant at Sta. 23 peaking (159,000 cells L^−1^) in a shallow chl a maximum (1.03 µg L^−1^; Station 23) found near the base of the mixed layer (50 m), this was also the depth for the biogenic silica maximum (240 nmol L^−1^) and the maximum >10 µm chl a concentration (0.46 µg L^−1^; Fig. 6). The %>10 µm chl a maximum (100% in the upper 20 m) corresponded to the surface *H. hauckii* maximum. This value declined with depth, but was still 46% at 50 m (*Mastogloia* and chl a maximum; Fig. 6). This pennate diatom maximum was weaker, but still present at Sta. 22 (Fig. 6) and contained single cells and aggregates of *Mastogloia* of up to 900 cells aggregate^−1^. Even though Station 22 and 23 were only ∼8 km apart, integrated cell abundance was 2–10 fold less at Sta. 22 (Fig. 6; Table 2).

**Table 2:** Station information, 0–60 m integrated data (m^−2^) and surface chlorophyll (µg L^−1^) from 2009.

Sta.	Lat. (°N)	Long. (°W)	H. hauckii (10^6^ cells m^−2^)	Rhizosolenia (10^6^ cells m^−2^)	M. woodiana (10^6^ cells m^−2^)	Chaetoceros sp. (10^6^ cells m^−2^)	R.intracellularis (10^6^ filaments m^−2^)	Calothrix (10^6^ filaments m^−2^)	Chl a(mg m^−2^)	>10 µm Chl *a* (mg m^−2^)	%>10 µm Chl *a*	bSi (mmol m^−2^)	Surface Chl *a* (µg L^−1^)
2	25.11	147.00	0	0	0.4	0	–	–	4.73	0.44	9.4	0.62	0.07
3	26.06	144.41	0	0	0.7	0	0.3	0.1	4.10	0.37	9.2	1.11	N/A
4	26.05	144.20	0	0	3.1	0	–	–	4.74	0.80	16.9	1.12	N/A
7	26.06	143.59	0	0	0.3	0	0.2	<0.1	1.20	0.15	12.8	0.99	0.08
8	26.06	143.48	0.3	0.3	0.4	0	–	–	3.82	0.27	7. 1	0.83	0.07
11	26.06	143.17	0.3	0	1.2	0	0.2	0	3.79	0.26	6.9	0.80	0.08
12	26.06	142.57	1.3	0	3.0	0	–	–	3.36	0.22	6.6	0.95	0.07
13	25.29	142.59	0	0	0.8	0	0.2	0	3.49	0.29	8.4	1.03	0.08
14	26.06	142.57	0	0	0.3	0	–	–					
15	26.05	142.35	–	–	–	–	–	–	3.76	0.24	6.4	1.09	0.07
16	26.06	142.15	–	–	–	–	–	–	4.58	0.39	8.4	1.07	0.09
17	26.06	141.53	<<0.1	0.1	2.3	0	–	–	6.75	0.47	7.8	1.15	0.07
18	26.05	141.33	0	0.1	1.6	0	–	–	3.51	0.27	7.8	1.01	N/A
20	27.30	141.59	0	0	0	0	–	–	4.35	0.39	8.9	1.15	0.06
21	23.29	140.00	0	0	3.4	<0.1	0.2	0	4.49	0.37	8.2	0.77	0.07
22	25.06	154.41	1.5	1.4	586	3.2	1.8	7.6	26.27	11.50	43.8	4.13	0.14
23	25.10	154.35	15.0	6.3	2954	6.0	3.7	10.7	26.70	16.63	62.3	7.32	0.15

Biogenic silica concentrations were the highest noted in 2009 and comparable to the values noted at the 2008 bloom station (Fig. 4, 6). Sta. 22 & 23 integrated biogenic silica concentrations (4.10 & 7.30 mmol m^−2^; Table 2) were ∼4–7 times higher than other 2009 stations but less than the maximum value (9.40 mmol m^−2^) noted in the 2008 cruise (Sta. 7).

### Cruise comparisons

**Figure 7:**
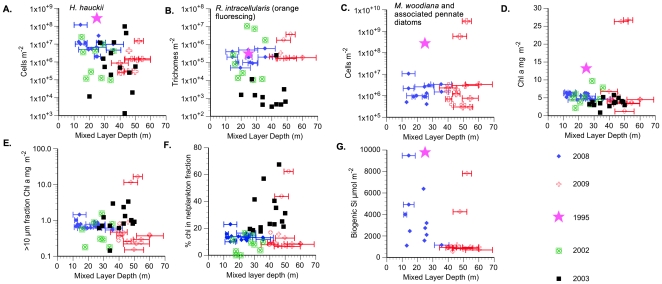
Mixed layer depth versus 0–60 m integrated biomass and abundance. Error bars represent the range of mixed layer depths observed in multiple CTD casts at individual stations. A. *H. hauckii*, B. Orange-fluorescing *Richelia intracellaris* trichomes (filaments) enumerated from 2 liter filtered samples, C. *Mastogloia woodiana* and associated small pennate diatoms, D. Total (>0.45 µm) chl a, E. >10 µm fraction chl a, F. % chl a in >10 µm size fraction, G. Biogenic silica (>0.6 µm fraction). See methods for 1995, 2002, and 2003 regional data sources.

The species dominance differences among the 2008 and 2009 blooms (Fig. 7) suggests that latitudinal floristic differences are superimposed on top of bloom dynamics. Floristically, high diatom abundance in all the blooms were associated with the shallow community identified by Venrick [37]. As fraction of the total, *Mastogloia* dominance in 2009 is clear at all but Sta. 13, whereas *Hemiaulus hauckii* was the dominant diatom in 2008 (Fig. 7). This general pattern holds even though the total cell numbers varied across orders of magnitude within years. Integrated cell counts from cruises in 1995, 2002 and 2003 provide context for *Hemiaulus* increases as well [2], [33]. Among the 5 cruises summarized, 26% of the observations exceed 10^7^ *Hemiaulus* cells m^-2^; only one of these stations was in 2009.

**Figure 8:**
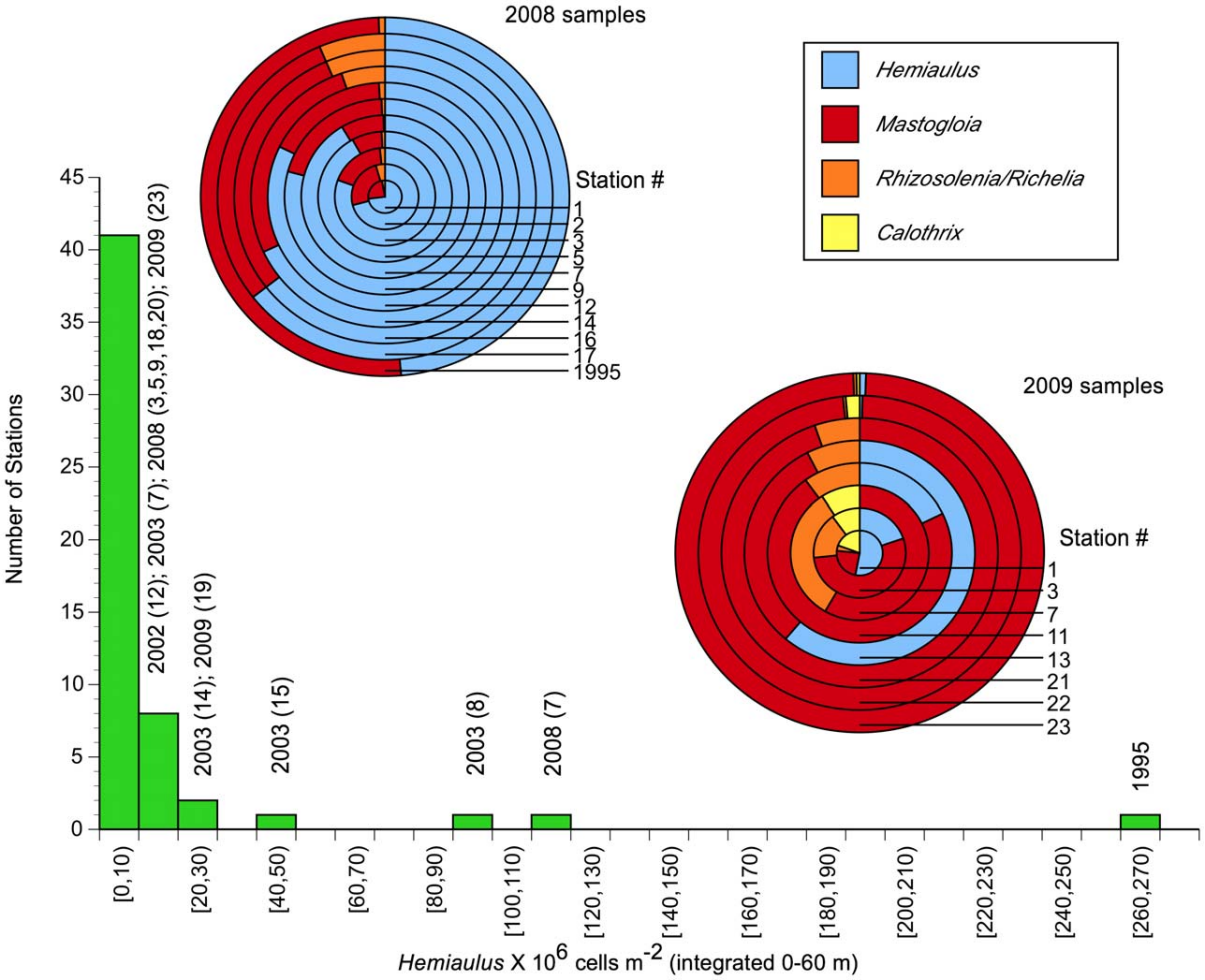
Species/abundance summary for the 2008/2009 blooms. Histograms express 0–60 m integrated abundance values for the DDA *Hemiaulus hauckii*. Individual columns are labeled with year (station number) for reference to the onion plots. The onion plots express the relative species abundance at stations for each year with each taxon color-coded (see Table 1, 2 for numerical data).

A comparison of mixed layer depth (MLD) between the 5 data sets provides no easy generalizations. The MLD in 2008 was generally ≤MLD in 2009 (Fig. 8) and was shallower than 62 m for all stations. The near surface salinity intrusions associated with the STF in 2008 created a complex structure with an isopycnal layer within the euphotic zone that spanned the upper 20–30 m but had vertical salinity structure within it. During the 2009 cruise, the MLD was 40–60 m with the deepest MLD at the bloom Sta. 22 and 23. These latter stations had a more typical isothermal and isohaline mixed layer structure observed in the NPSG during this time of year (Fig. 6). When the results of previous cruises from 1995, 2002 and 2003 [2], [33] are included, integrated cell abundance, chl a concentrations and bSi concentrations had no consistent relationships to MLD across multiple years (Fig. 7). *Hemiaulus hauckii* DDA abundance was highest when MLD was shallowest in 2008/2009, but while individual cruises can show a MLD relationship, such a relationship breaks down when examined using multiple years of data (Fig. 7). *Mastogloia* metrics (Fig. 7c) are clearly dominated by the 3 high abundance stations. The chl a -linked metrics (Fig. 7d-f) are dominated by the remarkably high values seen in the 25°N 2009 bloom. Biogenic silica concentrations have a primary and secondary maximum that are associated with different diatom species and two extremes of MLD (Fig. 7g). DDAs dominate the signal with a shallow MLD while *Mastogloia*-dominated stations occur with a deeper MLD.

## Discussion

The major sampling areas of the two cruises represent three time points in the summer chl blooms: early bloom (2009, 25°), post maximum bloom (2008) and post bloom termination (2009, 144°W). DDA increases were associated with the ocean color chlorophyll signatures in both years. Within the blooms, diatom abundance increased to 10^4^ cells L^−1^, and DDA development was maximal in the upper water column well above the nutricline. *Mastogloia* maxima at the same stations consistently occurred substantially deeper (40–60 m) at the base of or just below the mixed layer. Across all samples, the contribution of the >10 µm size fraction to total chlorophyll increased as diatom abundance increased and sharp gradients in cell abundance were noted over 10 s of km. Biogenic silica concentrations were highest within the chl a blooms and individual station maxima were generally associated with DDA or *Mastogloia* maxima. In both 2008 & 2009, the >10 µm size fraction increased substantially during blooms to well above the 10–15% norm. Based on the long-term sediment trap records at Station ALOHA, both blooms should result in substantial export production [17]. 

The two blooms diverged with respect to MLD (∼20 m in 2008; 50 m in 2009), the presence of the *Chaetoceros*-*Calothrix* DDA and the *Mastogloia*-dominated pennate community in 2009 but not 2008, and the pronounced shallow subsurface chl a maximum seen in 2009 compared to 2008. The physical setting of the two blooms differed as well; an isothermal and isohaline mixed-surface layer typical of the open sea in 2009 was contrasted with an isothermal shallow mixed layer vertically structured by salinity intrusions associated with the subtropical front in the 2008. These data, although limited, are the first deliberate sampling of ocean color chlorophyll features north of Sta. ALOHA in the NPSG and they show similarities and contrasts in the diatom speciation, biomass, and hydrography during these events.

### Biological community structure within bloom features

The 2008 & 2009 blooms contrast how diatom species composition, abundance, and chl a are linked within blooms seen in satellite ocean color data in the NPSG. The 2008 bloom (Sta. 7) had lower chlorophyll values than the 2009 25°N W bloom (Figs .2 and 7), was numerically dominated by *Hemiaulus hauckii* and *Mastogloia* was a minor component of the flora. Although the %>10 µm size fraction reach 40%, the >10 µm size fraction was insufficient to account for observed ocean color signature as has been noted in other satellite chl blooms [2]. In contrast, the 25°N 2009 bloom reached 0.25–0.4 mg m^−3^ at the surface with an unusual subsurface chlorophyll peak well above the nutricline. *Mastogloia* was the overwhelming numerical dominant although DDAs increased as well. The >10 µm chl a size fraction dominated (90–100%) at the surface and was sufficient to generate the satellite-observed chlorophyll value. The 2009 DCM chl a value (1.03 µg L^−1^) is rare for the open Pacific Ocean but is comparable to the highest chl a values observed in the region in the last decade (e.g. >1 µg L^−1^ Cyclone Opal [38], anticyclone near Sta. ALOHA [19]).

The limited field data cannot clarify whether the differences in species composition among blooms were the results of unique bloom types, stages in a regionally-consistent bloom development, or chance fluctuations. Data from the 2009 25°N bloom suggests *Mastogloia* may have an important role in creating the observed chlorophyll signature, but we have no way to determine if it occurs prior to the DDA maximum or is part of a unique regional bloom development. Regardless, the relatively low DDA abundance during the *Mastologia* peak in 2009 raises questions as to the nitrogen source supporting *Mastogloia* and the role that nitrogen fixation plays in creating these summer diatom blooms. It is possible *Mastogloia* contains a non-fluorescing symbiont that has not yet been identified.

The *Chaetoceros-Calothrix* was not seen in the 2008 bloom, the 2009 post-bloom at 26°N, nor was it observed in stations at ∼28°N [2], [39] or in Venrick’s [40] data from 28°N. However, it is commonly seen at Sta. ALOHA (R. Foster, per. comm). A floristic barrier may exist for this taxa between 25°N and 26°N in this region, although it occurs as far north as 33°45′N in the western Pacific Ocean [41]. Golden-fluorescing *Richelia intracellularis* was present at every station where samples were taken in both years, usually as a symbiont within in *Rhizosolenia clevei* or *H. membranaceus*. The notable lack of phycobilin fluorescence in the *H. hauckii* symbiont is puzzling. Previous work has considered these asymbiotic cells that had outgrown the symbiont as the result of nitrogen inputs [3], [42], as has been noted in *Rhizosolenia-Richelia* [43]. The *Hemiaulus* symbiont biology is more complex than previously recognized, although at this time we can only note the existence of this alternative fluorescence form. Methodologies for reliable detection and quantification are required.

*Mastogloia* spp. abundance in 2009 exceeded any value for the open sea known to us, and merits special consideration. *Mastogloia woodiana* (the dominant species) is a common member of the shallow phytoplankton community in the Pacific [40] and is frequently reported in association with the DDA community [2]. It can be a major contributor to carbon export at Sta. ALOHA [16], [44] and was a co-dominant species in DDA bloom sampled by the Brzezinski et al. [33]. *M. woodiana* often co-occurs with *H. hauckii* and has been reported as being epiphytically attached to chains of *Hemiaulus* and *Chaetoceros* [44], [45] where it may acquire N from the host diatom [41]. *Mastogloia* aggregates often contained coccoid cyanobacteria implying a direct association with remineralized N and P or association with possible diazotrophs in aggregate-generated microaerophilic environments. These aggregations shift the biomass of *Mastogloia* to the >10 µm size fraction as well as enhancing sedimentation rates [44]. Other aggregating pennate species have been reported [46] suggesting a linkage between diatom blooms and vertical transport that remains to be explored. The pennate flora of the open sea is poorly studied, but the consistent relationships to DDAs and high export potential suggests it is much more important than previous recognized.

### Physical conditions for bloom development and persistence

Our data provide a broad geographic context for evaluating factors that influence summer diatom bloom development in the NPSG. Our data are consistent with the pattern observed at time-series station ALOHA (22°45′N, 158°00′W) where chl a increases during DDA/diazotroph blooms following a shoaling of the MLD (<70 m) [6], but we did not find a relationship between MLD and diatom abundance as has been observed at ALOHA [5]. The high integrated chl a seen at Sta. 22 & 23 (2009) exceeded the maximum value (∼17.5 mg chl a m^−2^; MLD = ∼28 m) for summer blooms in the NPSG noted in Dore et al. [5] but occurred within the deepest MLD we observed (50 m). If those values are removed, then there is a general increase in 0–60 m integrated chl a in our data as the MLD shallows; however, the integrated chl values do not exceed 6.3 mg chl a m^−2^, a value less than the value of 8.7 mg m^−2^ used by Dore et al. [23] to define a bloom. It is not clear that the two data sets (ALOHA and STF blooms) have a common chl a or cell abundance basis for comparison at this time.

**Figure 9:**
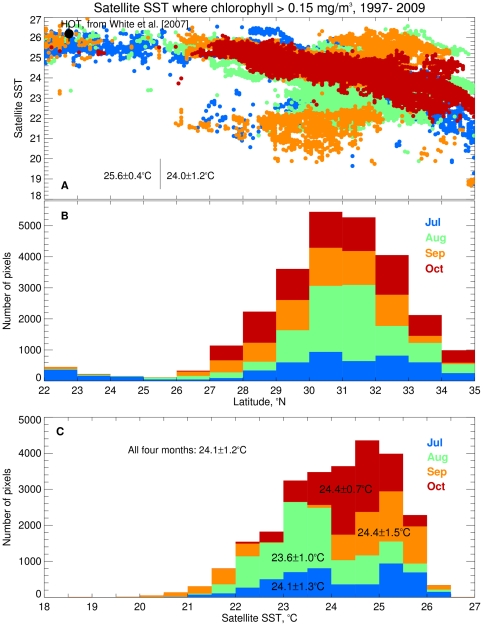
Temperature range for summer blooms detected by ocean color. A. Satellite sea surface temperature (SST) versus latitude presented as stacked bar graphs for areas with satellite chlorophyll >0.15 mg m^−3^ between 22–35°N and 160–130°W during July-Oct. for the years 1997–2009. B. Histogram of the prevalence of blooms against latitude. **C.** Histogram of the prevalence of the SST of the blooms. The data are color-coded by month: July data are blue, August is green, September is orange and October is red. In (A) the average and standard deviation of SST for blooms at Sta. ALOHA that were given in White et. al {, 2007 #22784} are shown by the black circle and error bars. Because of the large number of points (N = 26,302) in (A) not all of them are visible and the plotting order (July–Oct) makes points in Oct. appear more prevalent. The average and standard deviation of the SST south and north of 25.5°N is shown in (A).

White et al [6] concluded that DDA/summer chlorophyll blooms occurred only in a restricted temperature range of 25–27°C. Our observations at the sub-tropical front suggested that this range based on data from Sta. ALOHA is not applicable to the entire NPSG. We have plotted all ocean color summer chl blooms where chl>0.15 mg m^−3^ against the concurrent SST (Fig. 9). South of 25.5°N, the range seen at Sta. ALOHA (23–25.5°C) is only slight greater than White et al.’s [6] range. However, north of 25.5°N there is a decrease in the satellite SST associated with blooms as latitude increases. At the STF, most blooms occur between 22–26°C (Fig. 9c) with a suggestion of a July/August bimodal distribution at ∼23.5 and ∼24.7–25.3°C. Sta. ALOHA falls in the upper end of the observed range (Fig. 9) while blooms at the STF occur in the coldest waters. On a per-unit-area basis, blooms south of 27°N are numerically insignificant compared to the 30–32°N range (Fig. 9b). This does not capture the intensity of blooms, but does suggest that the area affected by blooms is dominated by the northern region. If the summer export production associated with diatom symbioses noted at Sta. ALOHA [17] is a general phenomenon, then the regions north of Hawaii up to the STF are likely a major source of export production as well. Since high abundance DDA events can occur without obvious ocean color signatures [2], Fig. 9 is capturing only a subset of the dynamics, but it is clear that the temperature range over which chl blooms occur is much wider than previously recognized.

Local hydrodynamics are also important in bloom dynamics. Fong et al. [19] concluded that mesoscale features are instrumental in DDA bloom formation. The proximity of the 2008 bloom to the STF and the associated SSH anomalies [47] is consistent with the role of mesoscale disturbances in creating DDA blooms (see also [4]). However, Wilson [22] linked the STF blooms to energy dissipation at the critical latitude while Calil & Richards [20], [23] invoke localized frontogenesis and associated upwelling in bloom formation. Our data indicates that the flora differs considerably in sampled blooms but we cannot resolve whether this is a temporal or latitudinal difference. However, it is apparent that the relationship of summer diatom blooms, DDA events and chlorophyll blooms to SSH anomalies is quite complex and floristic responses may be another important variable. Our data set does not permit evaluation of Karl et al.’s [17] suggestion that photoperiod plays a role in the annual cycle of symbiotic diatom export.

### Spatial variability in NPSG summer blooms

Summer DDA blooms clearly have high spatial heterogeneity and are not monotonic events within satellite-defined ocean color features. Venrick [40], Fong et al [19] and our study all found that cell abundance can change by multiple orders of magnitude over 10s of km. We suggest that these strong spatial gradients also reflect rapid temporal changes as well, and are manifestations of short-lived pulsed events and hotspots that are associated with the general regional features identified by ocean color data. The multiple day integrations of ocean color data often preferred for generating surface plots are likely blurring the episodic pulsing of these diatom events particularly if the events are spatially small.

The potential disconnect between the chl analysis and phytoplankton species abundance is evident from the 2008 STF bloom. This event did not reach the 0.15 µg L^−1^ bloom definition, yet DDA abundance in this bloom exceeded the very obvious 2009 25 °N bloom that exceeded the definition by nearly 5-fold. Watkins-Brand et al. [47] noted significantly lower N_2_ fixation rates in the 2008 bloom than a bloom farther south; it is not clear how general this trend is across other blooms and what the significance is to DDA biology.

### Summary

The bloom events in 2008 and 2009 were similar in terms of species composition, but had different dominant diatom species, biomass concentrations and physical water column characteristics. The high *M. woodiana* abundance in the 2009 bloom was unusual and was associated with unusually high chl a concentrations suggesting a larger role than previously suspected for this taxon. The ubiquitous co-occurrence of *Mastogloia* with DDAs suggests a tight linkage with N_2_-fixing diatom symbioses although the role of this pennate diatom in bloom development is still unclear. Size distributions were shifted towards the larger (>10 µm) phytoplankton size fraction in the blooms and bSi concentration increased up to 10-fold. Biomass in the >10 µm size fraction was inadequate to explain the chl a increase in 2008, but was greater than the satellite chl signature in 2009 where a non-DDA species dominated. Diatom blooms occur within the June–Oct. timeframe when mixed layers are <70 m [6], at temperatures between 21.5–26°C depending on latitude. Most blooms occur at intermediate temperatures north of 28°N. High spatial variability in abundance within blooms suggest that the broad general features observed in ocean color data are probably not homogeneous, but consist of numerous, short-lived diatom pulses. Summer diatom blooms in the NPSG have a complex relationship to mixed layer depth and mesoscale features and latitudinal species responses may superimpose additional complexity. Based on the long-term time series at Sta. ALOHA, these summer diatom blooms along the subtropical front should contribute substantially to export production.

## Supporting Information

Figure S1 **Transect 1, 2008: temperature, salinity, cell abundance, nutrients and chlorophyll** ***a*** **fluorescence.** **Stations are labeled above the figure.** A. Temperature (°C), B. Salinity (PSU). C. Silicate (µM) D. Phosphate (µM) E. Nitrate +Nitrite (µM) F. *R. intracellularis* (filaments L^−1^) G. *H. hauckii* (cells L^−1^), H. *M. woodiana* and associated pennate diatoms (cells L^−1^), I. CTD chl *a* fluorescence (µg L-1). J. Extracted chl *a* (µg L^−1^), K. >10 µm chl *a* (µg L^−1^), L. %>10 µm chl *a*, M. Biogenic silica total (nmol L^−1^). N. Cruise track with represented stations outlined in red. These figures are contoured data from the bloom transects identified in the Figure text.
(TIF)

Figure S2 **Transect 1, 2008 including the bloom Sta. 7.** Stations are labeled above the figure. A. Temperature (°C), B. Salinity (PSU). C. Silicate (µM) D. Phosphate (µM) E. Nitrate +Nitrite (µM) F. *R. intracellularis* (filaments L^−1^) G. *H. hauckii* (cells L^−1^), H. *M. woodiana* and associated pennate diatoms (cells L^−1^), I. CTD chl *a* fluorescence (µg L^−1^). J. Extracted chl *a* (µL^−1^), K. >10 µm chl *a* (µg L^−1^), L. %>10 µm chl *a*, M. Biogenic silica total (nmol L^−1^). N. Cruise track with represented stations outlined in red.
(TIF)

Figure S3 **Transect 2, 2008: Stations are labeled above the figure.** A. *H. hauckii* (Cells L^−1^, B. *M. woodiana* and associated pennate diatoms (cells L^−1^), C. *R. intracellularis* (filaments L^−1^), D. Extracted chl *a* (μg L^−1^), E. >10 µm chl *a* (μg L^−1^), F. %>10 µm chl *a*, G. Biogenic silica total (nmol L^−1^). H. Cruise track with represented stations outlined in red.
(TIF)

Figure S4 **Transect in the subtropical front, 2009 post-bloom.** Stations are labeled above the figure. Note x-axis is in longitude, not latitude. A. Temperature (°C). B. Salinity (PSU) C. Total diatom abundance (cells L^−1^) D. *R. intracellularis* (filaments L^−1^). E. Extracted chl *a* (µg L^−1^). F. >10 µm chl *a* (µg L^−1^). G. % >10 µm chl *a.* H. Biogenic silica (nmol L^−1^). I. Cruise track with represented stations outlined in red.
(TIF)
