# Supplementary figures and images for: Summer Diatom Blooms in the North Pacific Subtropical Gyre: 2008–2009
Source: PLoS One. 2012 Apr 6;7(4):e33109. doi: 10.1371/journal.pone.0033109 (PMC3320889; doi:10.1371/journal.pone.0033109)

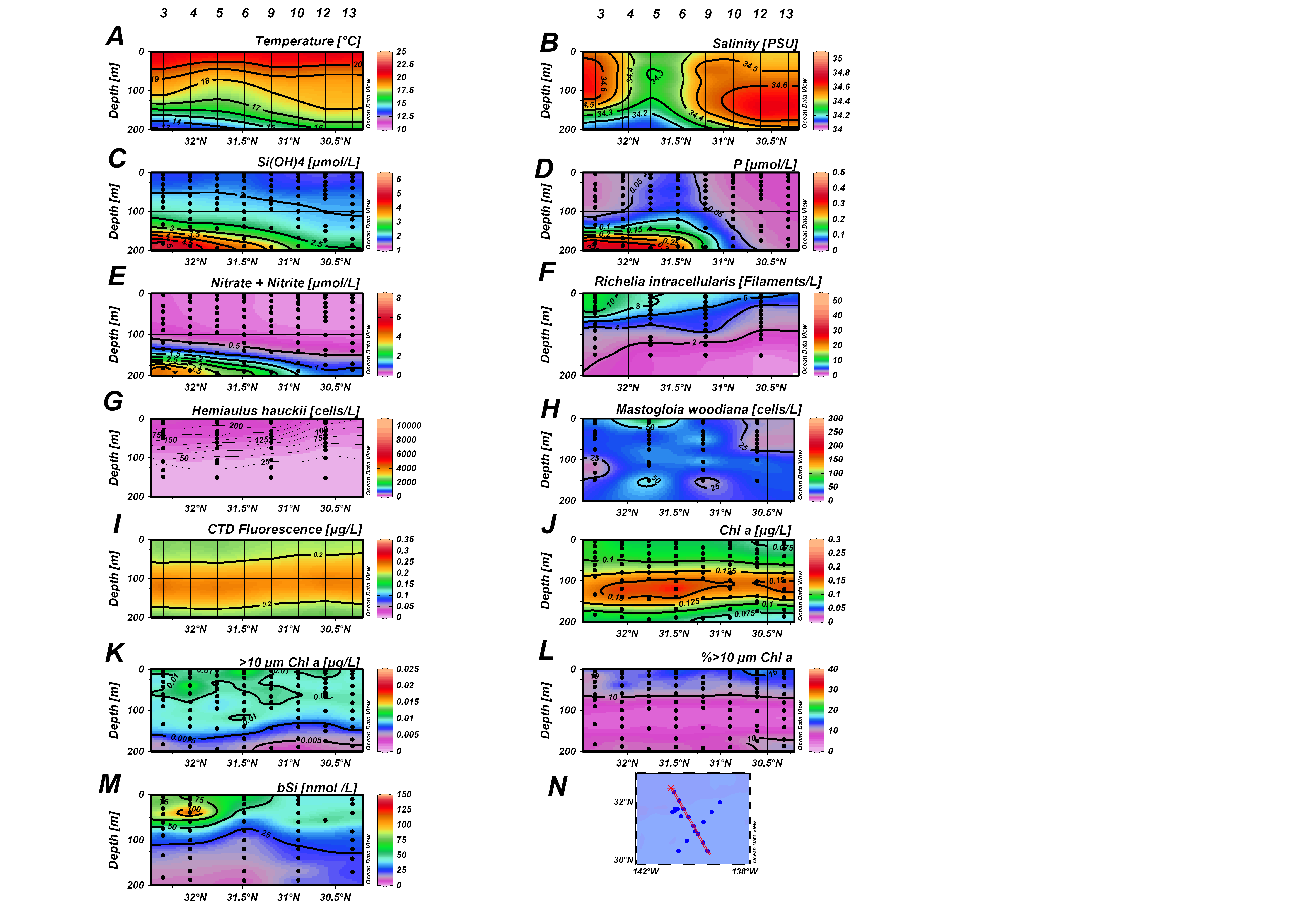

Supplement: Figure S1 — Transect 1, 2008: temperature, salinity, cell abundance, nutrients and chlorophyll a fluorescence. Stations are labeled above the figure. A. Temperature (°C), B. Salinity (PSU). C. Silicate (µM) D. Phosphate (µM) E. Nitrate +Nitrite (µM) F. R. intracellularis (filaments L−1) G. H. hauckii (cells L−1), H. M. woodiana and associated pennate diatoms (cells L−1), I. CTD chl a fluorescence (µg L-1). J. Extracted chl a (µg L−1), K. >10 µm chl a (µg L−1), L. %>10 µm chl a, M. Biogenic silica total (nmol L−1). N. Cruise track with represented stations outlined in red. These figures are contoured data from the bloom transects identified in the Figure text. (TIF) [file pone.0033109.s001.tif]

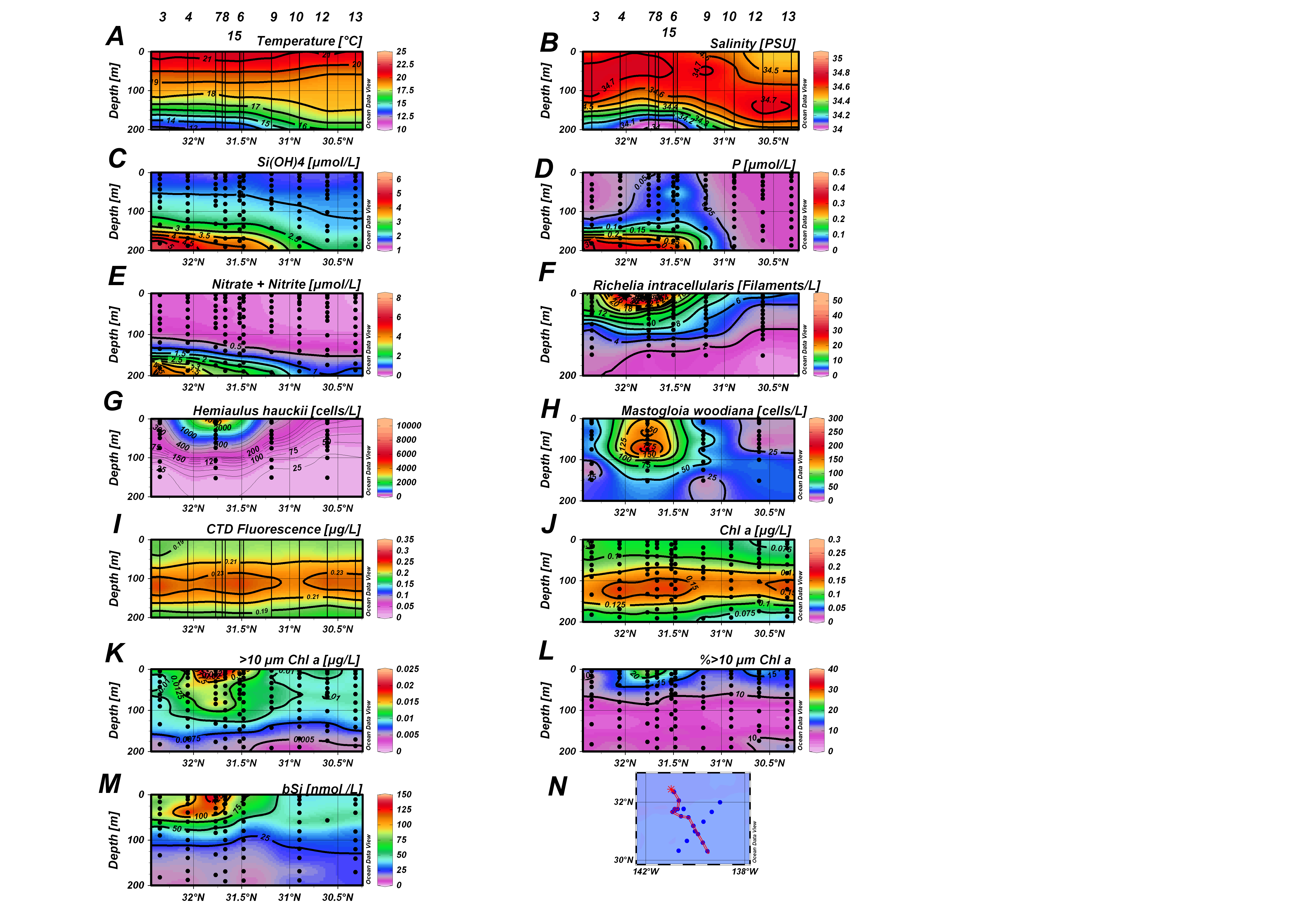

Supplement: Figure S2 — Transect 1, 2008 including the bloom Sta. 7. Stations are labeled above the figure. A. Temperature (°C), B. Salinity (PSU). C. Silicate (µM) D. Phosphate (µM) E. Nitrate +Nitrite (µM) F. R. intracellularis (filaments L−1) G. H. hauckii (cells L−1), H. M. woodiana and associated pennate diatoms (cells L−1), I. CTD chl a fluorescence (µg L−1). J. Extracted chl a (µL−1), K. >10 µm chl a (µg L−1), L. %>10 µm chl a, M. Biogenic silica total (nmol L−1). N. Cruise track with represented stations outlined in red. (TIF) [file pone.0033109.s002.tif]

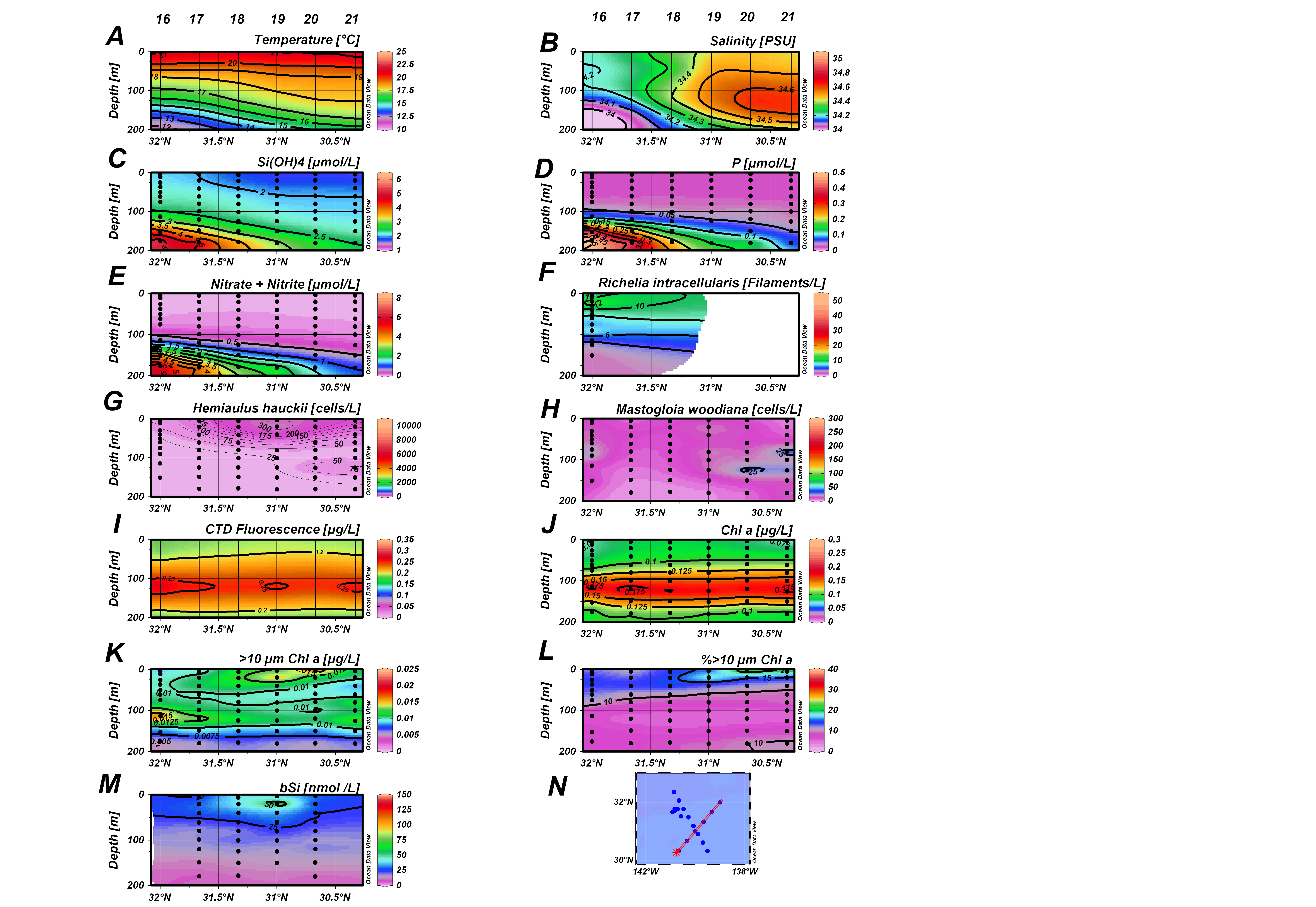

Supplement: Figure S3 — Transect 2, 2008: Stations are labeled above the figure. A. H. hauckii (Cells L−1, B. M. woodiana and associated pennate diatoms (cells L−1), C. R. intracellularis (filaments L−1), D. Extracted chl a (μg L−1), E. >10 µm chl a (μg L−1), F. %>10 µm chl a, G. Biogenic silica total (nmol L−1). H. Cruise track with represented stations outlined in red. (TIF) [file pone.0033109.s003.tif]

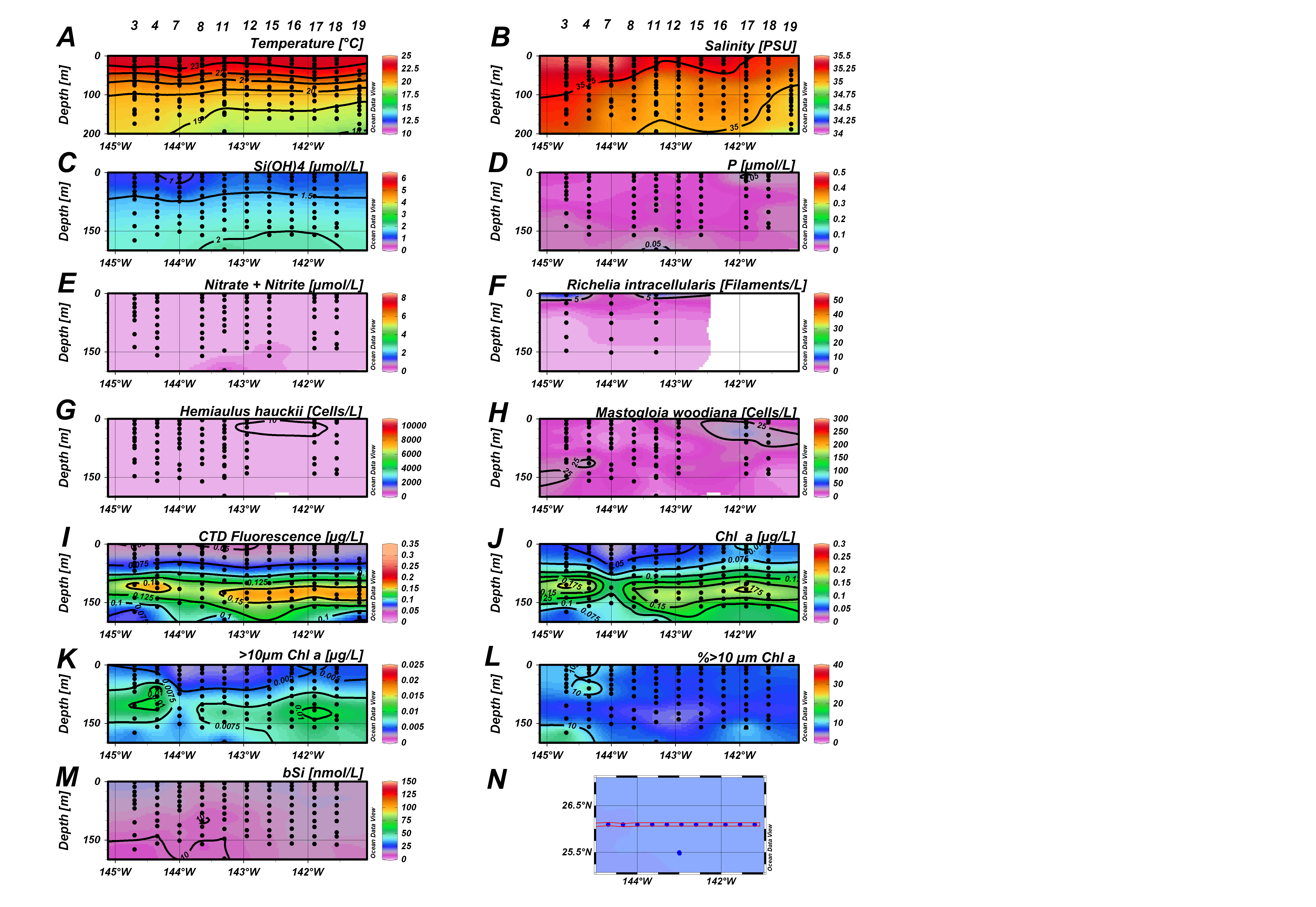

Supplement: Figure S4 — Transect in the subtropical front, 2009 post-bloom. Stations are labeled above the figure. Note x-axis is in longitude, not latitude. A. Temperature (°C). B. Salinity (PSU) C. Total diatom abundance (cells L−1) D. R. intracellularis (filaments L−1). E. Extracted chl a (µg L−1). F. >10 µm chl a (µg L−1). G. % >10 µm chl a. H. Biogenic silica (nmol L−1). I. Cruise track with represented stations outlined in red. (TIF) [file pone.0033109.s004.tif]
